# An Empirical Model for GaN Light Emitters with Dot-in-Wire Polar Nanostructures

**DOI:** 10.3390/mi11010082

**Published:** 2020-01-11

**Authors:** Jingyang Sui, Pei-Cheng Ku

**Affiliations:** Department of Electrical Engineering and Computer Science, University of Michigan, 1301 Beal Ave, Ann Arbor, MI 48109, USA; jysui@umich.edu

**Keywords:** gallium nitride, quantum confined Stark effect, strain control

## Abstract

A set of empirical equations were developed to describe the optical properties of III-nitride dot-in-wire nanostructures. These equations depend only on the geometric properties of the structures, enabling the design process of a III-nitride light emitter comprised of dot-in-wire polar nanostructures, to be greatly simplified without first-principle calculations. Results from the empirical model were compared to experimental measurements and reasonably good agreements were observed. Strain relaxation was found to be the dominant effect in determining the optical properties of dot-in-wire nanostructures.

## 1. Introduction

Group III-nitride semiconductors possess direct energy bandgaps spanning a wide wavelength range from ultraviolet (UV) and visible through near-infrared (NIR) [1]. Yet, efficient light emitters have so far only been realized in the 400–530 nm wavelength range [2]. The large lattice mismatch between III-nitride materials of different alloy compositions has led to issues of high defect density and large piezoelectric fields, which are attributed to the low radiative efficiencies. III-nitride nanostructures have been extensively studied to potentially overcome these issues. Among various nanostructures [3,4,5], dot-in-wire structures have shown great promises in improving light emitter efficiency in both UV and NIR wavelengths [6,7,8,9,10,11,12,13,14].

A dot-in-wire structure is a vertically oriented nanopillar, usually along the crystal c-axis, embedded with one or more disk-shaped heterostructures. Essentially, a quantum well in a conventional thin-film stack becomes a quantum disk in a dot-in-wire structure. In addition to improving radiative efficiency, dot-in-wire structures have been shown to exhibit unique optoelectronic properties that can be utilized for single-photon emitters with controlled polarizations and multicolor integration [15,16,17,18,19].

Both top-down and bottom-up processes have been used to fabricate these nanostructures. Due to effective lateral strain relaxation, the piezoelectric field in the quantum disk region is reduced which mitigates the quantum-confined Stark effect and increases the radiative efficiency. As the strain relaxation is not uniform in the radial direction and III-nitride semiconductors are piezoelectric, determination of the emission wavelength and other optical properties requires complex calculations involving strain field simulation and bandstructure calculations [20,21,22,23].

These quantum mechanical calculations significantly complicate the design process. The goal of this work is to derive a set of empirical equations that can describe the optical properties of dot-in-wire III-nitride nanostructures with reasonable accuracy via a small number of fitting parameters that can be easily obtained from simple optical measurements. We will focus on polar dot-in-wire structures, i.e., those grown vertically on a c-plane substrate. Polar III-nitride materials are generally easier to grow and achieve higher materials quality.

## 2. Methodology

### 2.1. Theoretical Model

**Figure 1:**
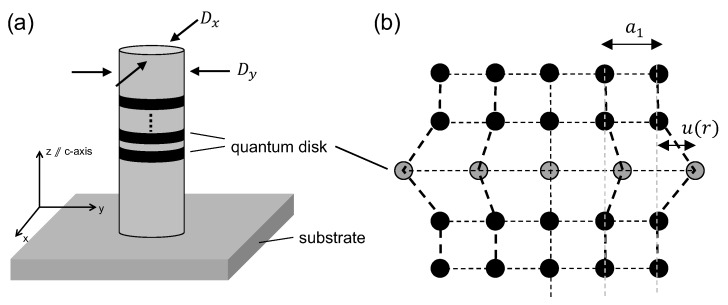
(**a**) The schematic of a dot-in-wire structure, as considered in this work. Multiple identical quantum disk regions are embedded in a nanopillar along the crystal c-axis. The quantum confinement is in the *z* direction. The nanopillar can have different dimensions in the *x* and *y* directions. If they are the same, we denote $D=D_{x}=D_{y}$. (**b**) The classical lattice model used in this work. The quantum disk region is modeled by a single-layer one-dimensional chain of lattice points. The lvariable u(r) is defined as the displacement of the lattice point with respect to the fully strained position. The quantum disk region is allowed to relax in the radial direction. The lattice points in the barrier are not allowed to move.

We consider dot-in-wire structures with a number of identical compressively strained quantum disks, as shown in Figure 1a. Rigorously speaking, both the barriers and the quantum disks are under stress. The barriers are under tensile strain, and the quantum disks are compressively strained. In this work, we will consider a much-simplified model, treating the quantum disks as one linear chain of lattices sandwiched between unstrained barriers as shown in Figure 1b [24]. We assume the number of quantum disks will only affect the results proportionally.

Under these assumptions, the lattice displacement profile after strain relaxation can be expressed as [24]

(1) $$u\left(r\right)=\left(a_{2}-a_{1}\right)\sqrt{\frac{k_{2}}{k_{1}}}\mathrm{sech}\left(\frac{\kappa D}{2}\right)\mathrm{sech}\left(\kappa r\right)$$

where *r* is the radial position of the lattice point; *D* is the diameter of the nanopillar; the subscripts 1 and 2 represent the barrier and the quantum disk region, respectively; $a_{1}$ and $a_{2}$ are the lattice constants; $k_{1}$ and $k_{2}$ are the spring constants used to model the elastic property of the material; and 1/κ is the characteristic length of strain relaxation in the quantum disk region which depends on the alloy composition of the quantum disk [21]. 1/κ signifies how elastic the nanopillar is upon strain relaxation. In Equation (1), we have assumed a circular nanopillar geometry. However it can be extended to an elliptical geometry by using two separate equations and $D_{x}$, $D_{y}$ and $r_{x}$, $r_{y}$ along the two ellipse axes, respectively.

### 2.2. Experimental Method

Our empirical model was verified by comparing to the experimental measurements. We prepared various dot-in-wire samples. The sample was grown by metal-organic chemical vapor deposition (MOCVD), consisting of a single InGaN quantum well and GaN barrier. After growth, the nanopillars were defined using a top-down process, first patterned by electron-beam lithography followed by reactive ion etching. Finally, the sidewalls of the nanopillars were made vertical by anisotropic etching in a diluted KOH solution. The diameters of individual nanopillars were then measured by scanning electron microscopy. For nanopillars with the same nominal sizes, the standard deviation of the diameter was found to be 1.5 nm. The optical properties (emission wavelength, optical intensity, and polarization properties) of individual nanopillars were measured by photoluminescence using a frequency-doubled Ti-sapphire excitation laser with a wavelength of 390 nm, pulse width of 150 fs, and repetition rate of 80 MHz. Optical emission from a single nanopillar within an array with 5 μm interpillar spacing was isolated using a confocal microscopy setup. Most optical measurements were performed at 10 K in this work, unless otherwise mentioned.

## 3. Results and Discussions

### 3.1. Emission Wavelength

From Equation (1), the hydrostatic compression strain along the radial direction can be expressed as

(2) $$\varepsilon \left(r\right)=\varepsilon^{\prime }\left[1-\mathrm{sech}\left(\frac{\kappa D}{2}\right)\cosh\left(\kappa r\right)\right]$$

where $\varepsilon^{\prime }$ is the strain in an equivalent thin-film III-nitride heterostructure. As the maximum strain occurs at the center of the nanopillar, it is expected that the bandgap shrinkage due to the piezoelectric field induced quantum-confined Stark effect is also the greatest. Hence electrons and holes are naturally confined toward the center of the nanopillar, suppressing the impact of non-radiative surface recombinations.

Of particular interests to us is the residual strain at the center (r=0) of the nanopillar:

(3) $$\varepsilon_{0}=\varepsilon \left(r=0\right)\equiv \varepsilon^{\prime }G\left(D\right)$$

where G(D) is a geometric parameter that depends only on the diameter of the nanopillar (or $D_{x}$, $D_{y}$ if we consider an elliptical nanopillar). As the nanopillar diameter decreases, the residual strain exponentially decreases, leading to an exponential change of the bandgap as a function of the nanopillar diameter. We can write the emission photon energy as a simple function of G(D):

(4) $$E_{ph}=E_{0}-B_{m}G\left(D\right)$$

where $E_{0}$ is the photon energy when the strain is completely relaxed and $B_{m}$ depends on the piezoelectric property of the material. Equation (4) has been previously verified experimentally at three different indium compositions [21,25]. The only fitting parameter required was 1/κ, as Equation (4) is derived solely from classical solid mechanics. The good agreement with the experiment suggests that the rapid decrease of the emission wavelength in a dot-in-wire structure when the diameter decreases is the result of strain relaxation governed by a geometric factor G(D), not due to quantum confinement. Indeed, a similar result was obtained using the effective mass theory, which took into account the strain relaxation and the quantum confinement [21].

### 3.2. Optical Intensity

Next, we consider the optical intensity. In a rigorous model, we need to calculate electron and hole wavefunctions at different eigenenergies and apply Fermi’s golden rule. For our objective, we approximate the band diagram along the vertical direction with a triangular potential well. It is known that the wavefunction Ψ(z) in an infinitely tall triangular well is given by an Airy function Ai(·) [26]:

(5) $$\Psi \left(z\right)=Ai\left[{\left(\frac{2m^{*}}{\hbar^{2}q^{2}F^{2}}\right)}^{1/3}\left(qFz-E_{n}\right)\right]$$

where

(6) $$E_{n}=-{\left(\frac{{\left(qF\hbar \right)}^{2}}{2m^{*}}\right)}^{1/3}z_{n}.$$

In Equations (5) and (6), *F* is the built-in electric field along the vertical direction, $m^{*}$ is the effective mass, and $z_{n}$ is the n-th zero of the Airy function. Although we need to sum over transitions between multiple conduction bands and valence band states, we first consider the ground state transition. At the same bias voltage, $F\propto \varepsilon_{0}$ for dot-in-wire structures with different diameters. We can write

(7) $$F\left(D\right)=F^{\prime }G\left(D\right).$$

Expanding the Airy function in Equation (5) in a Taylor series and keeping only the leading terms, we can calculate the overlap integral between the electron and hole wavefunctions to be

(8) $$\begin{array}{cc} \; & \int_{0}^{t}{\left(0.355-0.259z_{1}\right)}^{2}-F^{1/3}\int_{0}^{t}\left(0.355-0.259z_{1}\right)\left(0.259k_{c}z+0.259k_{h}\left(t-z\right)\right)dz\\ \; & +F^{2/3}\int_{0}^{t}0.259^{2}k_{c}k_{h}\left(t-z\right)dz \end{array}$$

where *t* is the thickness of each quantum disk and $k_{c,h}={\left(\left(2m_{c,h}^{*}q\right)/\hbar^{2}\right)}^{1/3}$. As transitions from other states will also contribute to the optical intensity, we can express the overlap integral η(D) hinted by the form in Equation (8) as

(9) $$\eta \left(D\right)=1+B_{1}G^{1/3}\left(D\right)+B_{2}G^{2/3}\left(D\right).$$

**Figure 2:**
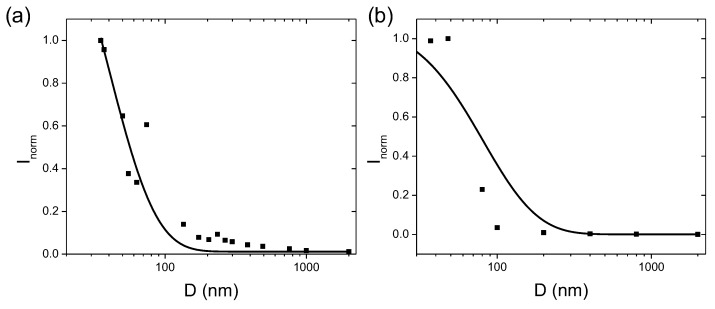
The comparison of the intensity model (solid lines) described by Equations (9) and (10) with experimental measurements (dots) for two samples. Both samples consist of a single InGaN quantum disk region in the nanopillar. The indium compositions are different in the two samples: In${}_{0.15}$Ga${}_{0.85}$N 3.5 nm and In${}_{0.32}$Ga${}_{0.68}$N 2.5 nm. 1/κ’s were previously obtained to be 14 nm and 31 nm for samples (**a**,**b**), respectively by fitting the emission wavelength data to Equation (4) [21]. The measured optical intensity (power received by the detector/quantum disk area) was normalized with respect to the value of the smallest nanopillar. One fitting parameter $B_{1}$ was used. $B_{1}=3.528$ and 0.6346 in (**a**,**b**), respectively.

To determine $B_{1}$ and $B_{2}$, we can prepare various arrays of nanopillars of different diameters lithographically defined on the same chip. We then measure the photoluminescence for each nanopillar array and normalize the intensities (power received by the detector/total nanopillar area) with respect to the value of the smallest nanopillar. We denote the normalized intensities as $I_{norm}$ and the normalized intensity of the largest nanopillar ($D=D_{ref}$) as $I_{ref}$. If $D_{ref}\gg 1/\kappa$, we can relate $B_{2}$ in terms of $B_{1}$ by $B_{2}=I_{ref}-\left(1+B_{1}\right)$. $B_{1}$ can now be obtained by fitting Equation (9) with the experimentally measured $I_{norm}$. In practice, once $B_{1}$ is obtained for a specific heterostructure, one can measure the intensity ($I_{meas}$) for a sufficiently large ($D=D_{meas}\gg 1/\kappa$) nanopillar and write the optical intensity for different diameters as

(10) $$I\left(D\right)=I_{meas}\frac{\eta (D)}{\eta (D_{meas})},$$

with only one fitting parameter $B_{1}$ required. Figure 2a,b compares Equation (9) to the experimental measurements of two dot-in-wire samples with different alloy compositions in the quantum disk region: In${}_{0.15}$Ga${}_{0.85}$N 3.5 nm and In${}_{0.32}$Ga${}_{0.68}$N 2.5 nm. Both samples consist of only one InGaN quantum disk embedded in the GaN nanopillar. The emission wavelengths of the original quantum wells were 476 nm and 654 nm, respectively at 10 K. 1/κ for both samples were obtained by fitting the emission wavelength data to Equation (4). Optical intensities were measured at the peak emission wavelength, at low temperature (T = 10 K) with a constant laser excitation power. Reasonable agreements between the theory and the experiment were observed for both samples even with only one fitting parameter $B_{1}$.

### 3.3. Emission Polarization

**Figure 3:**
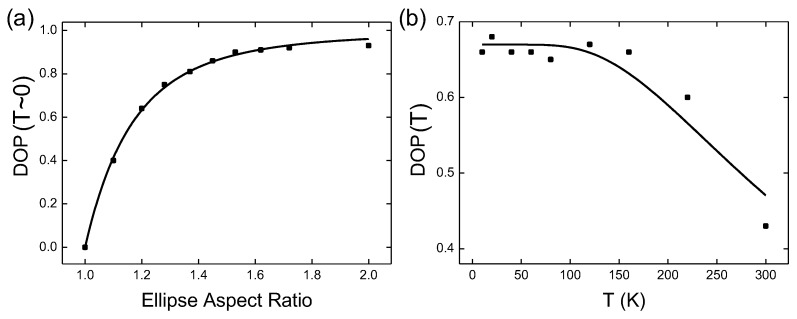
The comparison of the polarization model (solid lines) with experimental measurements (dots) for a sample with 1/κ=14 nm. It has the same epitaxial stack as sample (**a**) in Figure 2. The elliptical nanopillar has a short-axis dimension of 22 nm. (**a**) The DOP measured at T = 10 K for different ellipse aspect ratios under a low excitation condition (1 photon/pulse absorbed by the quantum disk). In (**a**), the fitting parameter α = 3.61. (**b**) The temperature dependence of DOP for the nanopillar with an ellipse aspect ratio of 1.2 under the same excitation condition as in (**a**). The fitting parameters are $E_{1}$ = 52 meV and $E_{1}$ = 58 meV.

Last, we look at polarization properties of the emission. Dot-in-wire elliptical nanostructures can emit polarized photons with a near unity degree of polarization (DOP) at a low temperature [16]. The nonzero DOP originates from the splitting of the valence band edge due to an asymmetric potential profile along the *x* and *y* directions. At low temperatures, all carriers occupy the ground states first. Hence, we can express DOP at a low excitation condition as

(11) $$\mathrm{DOP}\left(T∼0\right)=1-{\left(\frac{G(D_{x})}{G(D_{y})}\right)}^{\alpha }$$

where α is a fitting parameter. Figure 3a compares Equation (11) to the experimental measurements and a good agreement is obtained with a properly chosen α. The polarization direction is parallel to the long axis of the ellipse which we define as the *x* direction in the following discussions.

As temperature increases, or when the excitation is strong enough to generate carriers occupying higher-order states, the DOP is expected to decrease. We can use a three-state model to deduce the temperature dependence of the DOP. Suppose electron states do not contribute to DOP, while hole states can be categorized into the *x*-polarized state (with the lowest energy), *y*-polarized state, and non-polarized state (the higher-energy states), the carrier concentration in each state can be approximated by the Boltzmann distribution as follows:

(12) $$\begin{array}{cc} n_{x} & =n_{i}\exp\left(-\frac{E_{x}-E_{Fp}}{kT}\right)\\ n_{y} & =n_{i}\exp\left(-\frac{E_{y}-E_{Fp}}{kT}\right)\\ n_{np} & =n_{i}\exp\left(-\frac{E_{np}-E_{Fp}}{kT}\right) \end{array}$$

where $E_{Fp}$ is the quasi-Fermi level for the holes, and $n_{x}$, $n_{y}$, and $n_{np}$ are the carrier concentrations at the *x*-polarized, *y*-polarized, and non-polarized states, respectively. Note in Equation (12), the energy is the hole energy. Combining Equations (11) and (12), the DOP can be written as

(13) $$\mathrm{DOP}\left(T\right)=\left[1-{\left(\frac{G(D_{x})}{G(D_{y})}\right)}^{\alpha }\right]\left[\frac{1-\exp\left(-E_{1}/kT\right)}{1+\exp\left(-E_{1}/kT\right)+\exp\left(-E_{2}/kT\right)}\right]$$

where $E_{1}=E_{y}-E_{x}$ and $E_{2}=E_{np}-E_{x}$. Figure 3b compares Equation (13) to the experiment. Again, a reasonably good agreement was observed.

## 4. Conclusions

In summary, a set of simple, empirical equations that depend only on the geometric parameter(s) of a dot-in-wire structure grown vertically on a c-plane substrate: G(D)=1−sech(κD/2) have been developed and compared to experimental measurements. Although our model is not based on first principle calculations, reasonably good agreements can still be obtained with only one (or two for the temperature dependence of DOP) fitting parameter(s) required, which can be easily determined from a calibration sample consisting of a series of dot-in-wire structures of different diameters. These equations are expected to significantly simplify the design of III-nitride light emitters utilizing polar dot-in-wire structures. Our results also elucidate the important role of strain relaxation in determining the optical properties of dot-in-wire structures.
